# Analysis of factors influencing satisfaction with vocational rehabilitation services for young persons with disabilities in Sweden

**DOI:** 10.3389/fresc.2025.1573753

**Published:** 2025-05-30

**Authors:** Johanna Gustafsson, Ingrid Witte

**Affiliations:** ^1^ Department of Activity and Health, School of Health Sciences, Örebro University, Örebro, Sweden; ^2^ Centre for the Study of Professions, Oslo Metropolitan University, Oslo, Norway; ^3^ Disability Research, Örebro University, Örebro, Sweden; ^4^ Department of Social Work, School of Behavioural, Social and Legal Sciences, Örebro University, Örebro, Sweden

**Keywords:** vocational rehabilitation, service user satisfaction, supported employment, case management, person-centered rehabilitation, disability

## Abstract

**Purpose:**

The purpose of this study is to identify what factors influence user satisfaction with vocational rehabilitation services among service users in a Swedish context.

**Methods:**

In a randomized control trial, ordinal logistic regression was applied to a dataset of 631 completed questionnaires about the support provided in three different vocational rehabilitation programmes in Sweden—Supported Employment, Case Management and Regular Vocational Rehabilitation.

**Results:**

The factors Person-centeredness, Trust in Support Persons, and Experience that the activities help with getting a job were significant factors of satisfaction among service users. The ordinal logistic regression model explained between 34.3% and 49.9% of the variance in the material, depending on the pseudo R^2^-measure used.

**Conclusions:**

Service users who experience vocational support as person-centered, experienced trust in their support persons and that vocational rehabilitation activities help with getting a job are more satisfied with the vocational rehabilitation services than are other service users, independent of the vocational rehabilitation models used. Therefore, a person-centered approach is relevant to include in models’ development and service design of vocational rehabilitation.

## 1 Introduction

The labor market exclusion of people with disabilities is of great concern for both society and the persons themselves, and to improve labour market inclusion, government authorities and nongovernmental organizations provide a wide range of vocational rehabilitation services to people with disabilities. Vocational rehabilitation (VR) services are generally referred to as the process of supporting persons with illness or disability to obtain access to, maintain, or return to employment or other purposeful occupations (1). Research on VR programmes has focused predominantly on other quantitative outcomes, such as the percentage of successfully employed service users, to evaluate programme performance (2). However, in order to develop high quality VR services, it is necessary to understand the factors that influence service users’ experiences and satisfaction, where the former is based on what should happen during the VR process and whether it did, and the latter is based on whether expectations were met. Evidence from other settings, such as health care, suggests that high satisfaction is associated with increased treatment adherence and an increased likelihood of treatment completion (3, 4). In addition, without service users’ perspective, service evaluations may be biased towards the provider's perspective. Therefore, evaluating service user experience and satisfaction is a key factor in developing VR services in a desirable and efficient manner. Service user experience with VR has been explored in a number of studies, but user satisfaction as it relates to service quality has primarily been explored in the context of health services, such as mental health services and medical rehabilitation (3, 4) and has only received some attention in the area of VR (2).

### 1.1 The Swedish context

In Sweden, the Social Insurance Agency (SIA) has the responsibility to provide VR to young people (19–29 years) with disabilities who are eligible to receive activity compensation benefits due to reduced work ability, illness or impairment (5). The SIA guidelines advocate a person-centered VR approach where the service user's participation and active involvement in his or her rehabilitation is seen as a central prerequisite (6). Despite being a central prerequisite, it is not described how such an approach should be performed or secured in the VR process. Some elements that can be defined as person-centered are measured in SIA's annual user satisfaction survey. In the 2024 survey, a person-centered treatment by caseworkers (e.g., being treated with respect, understanding, engagement) received the highest satisfaction scores (65 out of 100) among the elements measured, but other elements that can be related to person-centeredness, such as caseworkers being easy to reach and providing information about the case, and the process being equitable and understandable received significantly lower scores (19 respectively 17 out of 100) (7). Thus, service users’ satisfaction with the agency's VR services remains in need of improvement if the services are to adhere to the advocated person-centered approach.

### 1.2 Vocational rehabilitation and service user satisfaction

A person-centered approach has increasingly become the approach favored in the provision of (vocational) rehabilitation (8). Briefly described, the person-centered approach aims to foster a partnership and the co-creation of care/rehabilitation between patients/clients and professionals regarding care or rehabilitation activities based on patients’/clients’ lived experiences by listening to the person's narratives and acknowledging patients’/clients’ resources and abilities to be an expert in their own life (9, 10). Research has shown that person-centered rehabilitation is associated with rehabilitation satisfaction (11, 12) and that satisfaction with services is, in turn, associated with commitment and completed treatment (3, 4).

The relationship between professionals and patients has been shown to be important for patient satisfaction (13) and research has highlighted the importance of this relationship, also named working alliance, as a means of implementing person-centered care and rehabilitation (14–16). Bordin [(17): 253] suggested that a working alliance includes “three main features: an agreement on goals, an assignment of task or a series of tasks, and the development of bonds”. The bond of attachment, developed in a relationship between those involved, is as important as mutual agreement upon goals and responsibilities in the tasks assigned. Thus, the quality of the working alliance is dependent both on the strength of the agreement about goals and tasks and on the strength of the attachment bond. He proposed that the working alliance is a necessary aspect of the change process and states that the working alliance “is one of the keys, if not *the* key, to the change process” [(17), p.252]. While a vast amount of research has been conducted on therapeutic relationships in psychotherapy, there is much less research on the impact of relationships in VR (18) even though studies pinpoint working alliances as important factors in VR interventions for people with mental illness [c.f. (18–20)] regardless of differences in types of professional roles or VR contexts (21). The relationship between the professional and the service user has been linked to a beneficial impact on employment outcomes in psychiatric rehabilitation programs (22, 23). Furthermore, service users who reach employment in VR (performed according to supported employment methods) are more likely to report a stronger working alliance than unemployed individuals are (24). However, the results are inconclusive, as others have found no overall association between working alliances and employment outcomes (25).

The performance of VR services is hence important for user satisfaction, and a review by Al-Rashaida et al. (2) revealed that counsellor characteristics, such as counsellor skills in listening, feeling empathy and giving support and encouragement, influenced user satisfaction with VR services. In addition, organizational factors such as well-organized services, qualified staff and involving service users in decision-making, as well as what actual vocational services were provided and their employment outcomes, were related to service user satisfaction. Service users who had positive employment outcomes also seemed to be more satisfied, but this relation was modified by job satisfaction.

A few qualitative studies have been performed in Sweden investigating service users’ satisfaction with VR. In a Swedish VR project, Andersén et al. (26) identified four themes related to users’ satisfaction: *opportunities for receiving various dimensions of support, good overall treatment by professionals, satisfaction with the working methods of the project, and opportunities for personal development*. These themes largely overlap with the themes identified by Al-Rashaida et al. (2). Another Swedish interview study revealed *professionals’ positive attitudes* to be one of three critical factors, alongside *experiencing hope and power* and *employing a holistic perspective and integrating (person-centered) mental health and vocational services* in the return-to-work processes of people with affective disorders (27). The professionals’ positive attitudes involved genuine interest and engagement, an understanding of the individual's needs and allowing the individual's needs to lead the intervention. Similar person-centered support approaches are also described by participants in supported employment interventions, where trustful relationships and diversified and individual support from the employment specialist were highlighted as important to the participants’ satisfaction with services (28).

Hence, a growing body of literature suggests that employing a person-centered approach influences VR satisfaction, where especially the quality of the relationship between professionals and service users seems to influence satisfaction with services. However, as the concept of user satisfaction is still to be defined in relation to the satisfaction of people with disabilities with VR programs (2), a more detailed understanding of what factors are associated with user satisfaction is thus important. Previous studies have mostly been qualitative, and to the best of our knowledge, no quantitative studies have explored factors related to satisfaction with VR from the service user perspective in different VR models. Such a horizontal perspective can advance the research field on user satisfaction by examining factors important to user satisfaction across different VR programmes. As it is reasonable to assume that user satisfaction enhances the efficiency of VR programs, knowledge about what contributes to user satisfaction can be seen as necessary to further develop, or sustain, the effective provision of VR services. In a Swedish context, Fogelgren et al. (29) studied the same population as used in this study and demonstrated rather modest differences (10 percentage differences at 18 months of follow-up) in employment success rates between the different VR programmes, and thus a deeper understanding of what features may influence programme effectiveness is needed to further develop these VR programmes.

### 1.3 Study purpose

The purpose of this study is to explore the factors associated with user satisfaction among young persons with disabilities across different VR programmes in a Swedish context. To explore this purpose, the following questions were asked: (1) What are the levels of user satisfaction for the three different vocational rehabilitation interventions? (2) What individual factors (age, sex, employment/internship/studies), contextual factors (type of intervention and help with completing the questionnaire), and relationship factors (trust in support persons and person-centeredness) are associated with satisfaction with services?

## 2 Methods

### 2.1 Settings

The material for this study was taken from a randomized controlled trial of three different VR interventions in Sweden, Supported Employment, Case Management or Regular Vocational Rehabilitation, carried out in regular activities in the support system in Sweden from November 2014 through December 2016 [Fogelgren et al. (29), provide more detailed information on the trial]. Ethical approval was granted by the regional Ethics Committee (Stockholm; dnr. 2014/1280-31/5).

In the trial, Supported Employment (SE) adopted a train-then-place model of VR (30). As outlined by Wehman (31), SE builds on the premise that persons with disabilities receive individual support by locating an appropriate job in an integrated setting, intensive job-site training, and permanent ongoing support. This support is provided by a qualified staff member (later called an employment specialist). In this study, SE services were provided by the Swedish Public Employment Services program “Special introduction and follow-up support”.

Case Management (CM) is an umbrella term for several different models of support for persons with severe mental illness with some common features. The original CM model was built on the principles that a single person, a case manager, is responsible for assessing needs, developing a care plan, arranging suitable care, and keeping contact with the person (32). In this study, the role of the case manager was specified to meet the conditions of the VR setting and the needs of the individual to improve opportunities in the labor market. Hence, the case manager was responsible for assessing VR needs, developing a VR plan, arranging suitable support, and keeping contact with the study participant.

Regular vocational rehabilitation (RVR) in the Swedish welfare system is a joint intervention by the SIA and the PES where these two authorities, together with the individual, chart the individuals’ needs and then plan VR activities accordingly. The activities can be either work preparation, such as counseling and wellness activities, or more work oriented, such as internships at workplaces (6). In this study, the RVR intervention was delivered as a collaboration between the SIA and the PES and was planned according to individual VR needs.

As described by Fogelgren et al. (29), the three interventions in the trial had some common features. All interventions were given in the context of VR, and the overall purpose was therefore that the services given should aim at entering, or returning to, employment or other purposeful occupations (such as studies). The interventions also had some distinct features. The RVR included broad but “nonintense” support, where the participants could receive support through the coordination of services as well as work preparation and training. However, RVR did not provide intensive workplace support, such as that provided in SE, or intense “whole life” support, such as that provided in CM. Therefore, the RVR could include a greater number of participants per caseworker than the SE and CM interventions. Like SE, the CM intervention provided intense support but had a broader support perspective, with intense “whole life” support (e.g., co-ordination of support for several life areas) rather than focusing on intense work support. The three models are distinguished from each other in several ways, as shown in Table 1.

**Table 1:** Characteristics of theVR interventions .

Intervention characteristics	Supported employment	Case management	Regular vocational rehabilitation
Aims and actions	Individualized work-focused support	Individualized “whole-life” support that can focus on medical/social issues, job preparations or be work-oriented/focused	Individualized support that can focus on medical/social issues, job preparations or be work-oriented/focused
Typical vocational service approach	Place-train	Place-train or train-place (participant decides)	Train-place
Services provided (the most common)	Job matching, follow-up support at workplace, coordination of support (to some extent)	Job matching, follow-up support at workplace, coordination of support	Job matching, follow-up support at workplace (to some extent), coordination of support
Primary professionals involved	Employment specialists	Case managers	Case workers
Intervention length	Length of the RCT, i.e., 3 years	Length of the RCT, i.e., 3 years	Length of the RCT, i.e., 3 years
Case load	Up to 20	Up to 20	Not specified
Minutes of support per week^a^	60	83	27
Number of contacts/week^a^	1.05	1.95	0.9
“Experiencing that the support person has enough time for you”^b^	58%	70%	50%
Time spent talking about work (min/week)^a^	44.2	34.1	16.2
“Talking about work with your support person”^b^	97%	97%	81%
Time oriented towards coordination of support, social/medical (min/week)^a^	2.62	11.56	2.09
“We have been working on my health”^b^	81%	92%	82%
“We have been working on my social relations”^b^	65%	81%	59%

^a^ <21 weeks into intervention), assessed by the primary professionals.

^b^ As reported in the participant survey. Source: A randomized evaluation of interventions for young people with disability pension—Social Insurance Report 2017:5 (33).

### 2.2 Participants

In the trial, 1,063 participants with activity compensation (eligible for individuals with reduced work ability due to illness or impairment), 19–29 years of age, were randomized into one of the three different VR interventions (i.e., Supported Employment, Case Management or Regular Vocational Rehabilitation). Informed consent was obtained from all individual participants included in the study. A total of 59% (631/1,063) of the participants in the randomized controlled trial answered the questionnaire. Of the persons receiving CM the response rate was 73%, but of the persons receiving SE and RVR the response rate was approximately 50%. According to a previous report from the trial describing the participants (33), the participants who did not answer the questionnaire were more likely to have at least a secondary education or had daily activities (i.e., sheltered workshops) according to the Swedish Act concerning Support and Service for Persons with Certain Disabilities (34). Apart from this, the participants who responded to the questionnaire did not significantly differ from the total trial population.

### 2.3 Material

At the 6-month follow-up in the randomized controlled trial, the participants were encouraged to complete a questionnaire about how they perceived VR support. The questionnaire consisted of questions about the overall support (8 items, e.g., How do you feel about the support you received?) and questions about specific parts of the support. Of the latter, 16 items targeted person-centeredness, i.e., co-creation of VR activities [e.g., Have you and your support person(s) planned together what you will do in this process?], working alliance [e.g., Do you and your support person(s) agree on your goals?], and dialogue with the support person [e.g., Do your support person(s) listen to what you need help with?]. 9 items were related to support for work [e.g., Can your support person(s) help you keep a job?] and 8 items were related to support for study (e.g., Did the support you received from your support person(s) help you study? In addition, 4 items were related to the benefits of participation (e.g., Did you get a job or internship in a workplace?). Theoretically, the questionnaire builds on the frameworks of person-centered rehabilitation (9, 10), working alliance (17, 19, 24, 25) and work inclusion (30, 31). Some questions were reported on a nominal scale, others were reported on a three-level ordinal scale, and some questions were reported on a four-level ordinal scale. In addition to questions about the overall support and outcomes of the support, information about the type of intervention, gender, age and whether the participant had received help completing the questionnaire was collected.

### 2.4 Data analysis and measures

All the data analyses in this study were performed in SPSS version 28.

To describe the general characteristics of the participants in the dataset and overall satisfaction with VR, descriptive statistics with frequency counts and percentages were used for the ordinal and nominal variables, while means and standard deviations were calculated for the continuous variables. To analyze if there were differences in the level of satisfaction for the three different interventions, a Kruskal‒Wallis test was performed, and to further explore the significance of the differences shown by the Kruskal‒Wallis test, *post hoc* Mann‒Whitney *U*-tests were performed to compare the three intervention groups. (See Supplementary Material 1 for figures.)

To examine what factors were associated with satisfaction with VR services among service users, ordinal logistic regression was used as the most suitable option (35), and the proportional (or cumulative) odds model (36) was used in this study.

The *dependent variable* in this study was an item in the questionnaire measuring satisfaction with overall support for VR. The item measured satisfaction with overall support on a three-level ordinal scale with the alternatives “good”, “neither good nor bad” and “bad”. Reasons for choosing a three-level scale included clinical experiences of a three-level scale being easier to understand for the study population than a scale with more levels.

The *independent variables* in this study were the background variables Gender, Age, and Help (to complete in the questionnaire), as well as variables related to previous research on satisfaction in VR; Type of intervention, Having been employed/internship/studies during the VR, Experience that VR activities help with getting a job, Trust in support persons, and Person-centeredness. See Table 2. These variables were selected because previous research indicates that they are important in vocational rehabilitation and work inclusion of persons with disabilities (2, 19, 21, 24–27).

**Table 2:** Independent variables, used in the correlation analysis.

Independent variables	Type	Fulfills POA
Gender	Nominal	Yes, sig. 0,719
Age	Continuous	Yes, sig. 0,310
Help with filling in the questionnaire	Nominal	Yes, sig. 0,027
Type of intervention	Nominal	Yes, sig. 0,089
Employment/Internship/Studies during VR	Nominal	Yes, sig. 0,039
Experience that the activities help with getting a job	Ordinal (treated as continuous)	Yes, sig. 0,092
Trust in support persons	Nominal	No, sig. 0,006
Person-centeredness	Continuous (index)	Yes, sig. 0,803

POA, proportional odds assumption.

The independent variable “Person-centeredness” was created as an index consisting of six items, all of which are theoretically related to the concept of person-centeredness (i.e., goal agreement, task assignment, (support) bonds [(15): 253] and counselor skills (10, 24). The items were highly correlated in the analysis, where Cronbach's alpha for the whole index was 0,859, well exceeding the recommendation of 0,7 (37). The items in the index were reported on a four-point ordinal Likert scale and were treated as continuous variables to form an index. The individual items that form the index, the properties of the items, and the whole scale are shown in Table 3.

**Table 3:** Index “person-centeredness”.

Items in index “person-centeredness”	*N* ^a^	Item mean	Item std. deviation	Scale mean if item deleted	Scale variance if item deleted	Cronbach's *α* if item deleted from index
Do your support persons listen to what kind of help you want?	490	2,74	0,584	12,9	7,229	0,825
Are your support persons easy to get in touch with?	490	2,64	0,668	12,99	7,108	0,836
Do your support persons have enough time for you?	490	2,57	0,692	13,07	6,965	0,834
Are your support persons giving you the information you want?	490	2,67	0,632	12,97	7,128	0,829
Have you and your support persons planned together what you will do in the intervention?	490	2,49	0,744	13,15	7,004	0,849
Do you and your support persons agree on your goals?	490	2,53	0,762	13,11	6,704	0,837

^a^ The number of respondents to each item in the index is less than that for the whole questionnaire due to internal missing answers.

The multicollinearity of the independent variables was tested with the variance inflation factor (VIF). The 8 independent variables in this study had VIFs between 1,006 and 1,491 (see Supplementary Material 3 for figures), indicating no problematic issues with high multicollinearity within the independent variables.

## 3 Results

Two research questions guided this study. For the first question, i.e., what are the levels of user satisfaction for three different vocational rehabilitation interventions, nonparametric tests were performed to compare user satisfaction in the three intervention groups. Participants in the randomized controlled trial were generally satisfied with the VR provided during the trial, with 71% responding that the support received was good and only 4.3% of participants responding that the support received was poor, while the remainder (24.7%) found the support quite adequate See Table 4.

**Table 4:** Satisfaction with the support, participants characteristics, participants in the interventions, outcomes of participation, and participants’ experiences of the support.

Variable		*N*	Valid percent	Mean	Std. Dev.	Min.	Max.	Valid	Missing	Total
Satisfaction with support	Bad	26	4,3							
	Neither good nor bad	150	24,7							
	Good	431	71							
								607	24	631
Gender	Female	316	50,1							
	Male	315	49,9							
								631	0	631
Age		631		25,72	2,58	19	31	631	0	631
Help with the questionnaire	No	423	69,9							
	Yes	182	30,1							
								605	26	631
Intervention	RVR	158	25							
	CM	287	45,5							
	SE	186	29,5							
								631	0	631
Employment/Internship/Studies	No	152	24,6							
	Yes	465	75,4							
								617	14	631
Activities help with getting a job		534		2	0,956	0	3	534	97	631
Trust	No trust	11	1,8							
	Partly trust	106	17,2							
	Trust	501	81,1							
								618	13	631
Person-centeredness		490		2,606	0,523	0	3	490	141	631

However, the three intervention groups differed from each other, where persons receiving CM were significantly more satisfied with the support than persons receiving SE were, who in turn were more satisfied with the support than persons receiving RVR were. The differences between the rank totals of 331,10 (CM), 299,22 (SE) and 257,88 (RVR) were significant, *H* (2, *n* = 607) = 27,3, *p* = <,001 (See Table 5).

**Table 5:** Satisfaction with support, differences between the VR interventions.

A. Kruskal‒Wallis test.			
Kruskal‒Wallis test			
Variables	Intervention	*N*	Mean rank
Satisfaction with support	RVR	148	257,88
	CM	283	331,10
	SE	176	299,22
	Total	607	

B. Grouping variable: intervention.	
Method	Satisfaction with support
Kruskal‒Wallis H	27,319
df	2
Asymp. Sig.	<,001

For the second research question, i.e., what factors are associated with satisfaction with services, a correlation analysis was performed to determine whether individual, contextual and interactional factors were correlated with service user satisfaction. According to the full ordinal regression model, when examining which factors were associated with user satisfaction across the interventions (with all the independent variables chosen), the interventions *per se* lost their significance as a factor influencing satisfaction with vocational rehabilitation (see Table 6). Instead, the variables on the interactional level; “Experience that the activities help with getting a job” [95% CI (0,485, 1,121)], “Person-centeredness” [95% CI (1,374, 2,593)], and “Trust in support persons” [95% CI (−2,182, −0,801) for “Partly trust” as Trust was set as the reference point] were the significant variables influencing user satisfaction with vocational rehabilitation. The factors at the individual level as Gender [95% CI (−0,309, 0,766)] and Age [95% CI (−0,163, 0,044)] were not significant. Neither were the factors at the contextual level, i.e., Intervention [RVR 95% CI [−1,277, 0,258], CM 95% CI [−0,269, 1,076] and SE was set as the reference Point], Employment/internship/studies [95% CI (−0,906, 0,385)], and Help with the questionnaire (95% CI [−0,509, 0,712).

**Table 6:** Full ordinal logistic regression model.

Variables		Estimate	Std. error	95% confidence interval		Wald	Sig.
				Lower bound	Upper bound		
Satisfaction	Quite alright—threshold	0,164	1,572	−2,917	3,246	0,011	0,917
	Good—threshold	3,914	1,631	0,718	7,109	5,76	0,016
Gender	Male	0,228	0,274	−0,309	0,766	0,693	0,405
	Female	0 ^a^					
Age	Age	−0,059	0,053	−0,163	0,044	1,251	0,263
Help with the questionnaire	No	0,102	0,311	−0,509	0,712	0,106	0,744
	Yes	0 ^a^					
Intervention	RVR	−0,484	0,379	−1,227	0,258	1,633	0,201
	CM	0,404	0,343	−0,269	1,076	1,384	0,239
	SE	0 ^a^					
Employment/internship/studies	No	−0,26	0,329	−0,906	0,385	0,625	0,429
	Yes	0 ^a^					
Activities help with getting a job		0,803	0,162	0,485	1,121	24,537	*0,000* ^b^
Trust	No trust	−0,195	1,012	−2,178	1,788	0,037	0,847
	Partly trust	−1,491	0,352	−2,182	−0,801	17,912	*0,000* ^b^
	Trust	0 ^a^					
Person-centeredness		1,983	0,311	1,374	2,593	40,681	*0,000* ^b^

^a^ This parameter is set to zero because it is the reference point.

^b^ Significant at *p* = 0,00 level.

According to the most conservative measure of the ones tested, the model explained 34,3% (McFadden pseudo R^2^), and according to the most liberal of the measures tested, the model explained 49,9% (Nagelkerke pseudo R^2^) of the variation. Moreover, the full model fulfils the assumption of proportional odds with the test of parallel lines with a significance of 0,136. However, due to missing data points, only 427 out of 631 patients could be included in the full ordinal logistic regression model.

## 4 Discussion

### 4.1 Main findings

The purpose of this study is to explore service user satisfaction across three different VR interventions, and to determine whether individual, contextual and interactional factors were correlated with satisfaction. The overall results suggest that VR participants who experience that they are given entry point opportunities for person-centered support in the VR rehabilitation process, who experience trust in their support persons and who experience that the activities in the VR process will help with getting a job are more satisfied with the services than other participants are. The significant difference in satisfaction between the three models of VR that was apparent without the ordinal logistic regression model disappeared when other important factors, such as the levels of person-centeredness, quality of working alliance, and goal-attainment, were taken into account. Thus, the specific model of VR was no longer important, but rather the different elements of the model.

The elements common to the three VR models are first and foremost individualized support focused on the participant's needs and the coordination of support to help participants navigate and access different services and supports. However, the goal of the latter may differ between the VR models, where CM and RVR coordination support is aimed at facilitating the participant's daily life, while the coordination goal of SE is more focused on supports and services that facilitate entry into the labor market. A third common element for the VR models in this study was an overarching goal of helping the participant enter the labor market. All of the VR models have this focus to some extent, but the strength of the focus can vary between VR models, as can the definition of the labor market. In SE a rapid entry to the regular labor market is an important principle, and so is paid work (30, 31), while the other VR models may not apply rapidity and have a less strict definition of the labor market and paid work, including for example internships and sheltered work. As the survey did not define what type of work (paid job or unpaid internship) the activities in the VR process were intended to help participants obtain, there may be different representations of work among participants that influence their experiences of goal attainment in terms of obtaining a job. However, despite the differences between VR models in terms of coordination goals and definitions of entering the labor market, both individualized support, coordination support, and labor market focus are elements that should be seen as constituent parts of the VR models.

The finding that the specific model is less important than its elements aligns with findings in other fields, such as psychotherapy, where research indicates that the specific factors associated with various methods have limited explanatory value regarding the methods’ efficacy and the identification of what helps. Instead, as for example Wampold et al. (38), suggest that common factors found across the board, regardless of theoretical approach or technique, may be of greater importance. Previous studies of user satisfaction in VR have highlighted the influence of the actual vocational services model to a greater extent (2, 26, 27). However, as several of these VR models build upon a person-centered approach, the results of this study are not contrary but rather are in line, as the factors found to be important for satisfaction in this study might be more easily achieved in a VR model where there are good conditions for providing person-centered services. As, for example, SE methods, where the organizational conditions with a smaller case load in combination with a clearly stated principle that the service is to be designed according to the individual's wishes and needs should provide professionals with real opportunities to focus on entry-level person-centered support in the process and participants’ influence over services (39, 40).

Theoretically, it could thus be argued that the SE intervention should have been the VR intervention with the most satisfied users, as the method should focus on employment and social inclusion in the regular labor market (39), in combination with a person-centered approach aiming to create a working alliance with the user (40, 41). Instead, the most satisfied users were among those who received CM intervention; this support model theoretically has a strong focus on partnership and trustful relationships but does not have a specific work focus (28). However, in this trial, the CM intervention had a work focus, but the intervention given was substantially less work oriented (i.e., time spent talking about work) than the SE intervention and instead focused on a broader scope, encompassing support regarding health and social issues to a substantially greater degree than the SE intervention (29). Hence, in practice, participants may experience “whole-life” support, where the support also targets other challenges that impact successful employment, as much, or more, of importance for the experience of satisfaction with services than a stronger focus on work support and less on support with health and social issues.

User satisfaction may also be influenced by the experienced timeliness of support and by what individuals themselves consider their most important needs for the time being and how their needs are experienced as related to each other. As many participants may face multiple work-related challenges and barriers, e.g., related to their health, social situation and employability, a whole-life support approach may seem to be the most fair and feasible approach because the challenges and barriers are most likely intertwined and hence need to be addressed as such. The importance of VR methods for addressing several life areas, such as work support and mental health simultaneously, has been found in previous Swedish studies on users’ experiences with VR (26, 27). A “whole-life” support is also well in line with the person-centered approach with its starting point in individual situations and needs.

In the trial, the CM intervention was found to be the most intense support intervention (29), which may have influenced the higher user satisfaction. The intensity of the support, i.e., the time allocated to support, has been shown to be important for the construction of a work alliance. Topor et al. (42), found that both quality and quantity of time matter for work alliances in psychiatry interventions, where quantity of time relates to the experience of having more time during, between, and after the sessions than expected. The quality of time related to having focused time together and to the sense of being a “real-life” person, who was in the professional's thought between the sessions and where the timing followed the person's needs. The quality of the working alliance has also been shown to be influenced by the attitude of the professional (case manager) and the practical support they offer (43). Both the quantity and quality of time and practical support are to some extent captured in the person-centeredness index used in this study and as seen in the results, associated with user satisfaction. Hence, support persons, who put effort into creating a person-centered working alliance with their service users are likely to also have more satisfied users.

Another interesting result in this study is that whether the participants were or had been in employment, internship or studies during the intervention did not seem to affect the level of satisfaction with the services on any significant level. This finding is inconsistent with previous findings where positive employment outcomes, if also combined with job satisfaction, seem to lead to better user satisfaction (2). In this study, there were no observations of the influence of job satisfaction on overall satisfaction with support, but employment outcomes were greater for the SE intervention than for the CM intervention and the RVR intervention, although with rather moderate differences of approximately ten percentage points (29). However, as these employment outcomes were measured after 18 months and through the use of a questionnaire after only 6 months, the correlation between employment outcomes and user satisfaction might have been weaker at the beginning of the interventions, when fewer employment outcomes were reached. The association between user satisfaction and the experience that the activities in the VR process help with getting a job may be related to the timing of the questionnaire, where expectations to achieve a successful employment outcome in the near future may still be high after 6 months into the VR process, especially if the service users experience trust in their support persons and their supportive behaviors.

The experience of a high level of person-centeredness in the VR process may also relate to whether the participants themselves have decided on the tempo in the process where principles of rapid job search and rapid job entry, as emphasized in the European Union of Supported Employment (44), may not be entirely in line with the individuals’ wishes and needs, at least not from a short-term perspective. That service users have different preferences and needs related to the rapidness of job entry is something that employment specialists in VR have highlighted (45), as are gendered preferences in relation to tempo in VR services, where women prefer a slower process of job entry than men (28). This preference could have altered the employment outcomes in the trial, as men were more likely to have reached employment in all interventions after 15 months, with the largest sex differences demonstrated in the SE intervention, followed by the CM intervention (33). These sex differences were less significant after 18 months of age (29). However, for user satisfaction with services, there were no significant sex differences in which factors men and women relate to satisfaction with VR services, suggesting that a person-centered approach and trustful relationships in VR meet the needs and preferences of both men and women, irrespective of what needs and preferences are or the gendered influence on VR outcomes.

### 4.2 Strengths and limitations

This study has several strengths and limitations that should be mentioned. For the latter, a central limitation is the lack of data on method fidelity in the interventions. The results regarding satisfaction with the specific interventions should therefore be interpreted with that in mind, as there are no data on to what extent the interventions followed the fidelity criteria for each method. However, the factors examined across the methods were quite specific and guided by a theoretical framework on person-centeredness as well as by previous empirical findings. This could be considered a strength, as earlier research highlights that the concept of person-centered rehabilitation suffers from a lack of detail and clarification, despite the growing recognition of person-centeredness as an essential component of rehabilitation quality (46). Another limitation is the cross-sectional design and the timing of the questionnaire in relation to some of the questions asked, as satisfaction with services may fluctuate over time, depending not only on employment outcomes (23, 24) but also on fluctuating health conditions (such as mental illness) (24). Regarding trust in the support person and partnership, the timing of the questionnaire is more appropriate, as De Leeuw et al. (43) indicated that working alliances are established in the first 3 months of a patient–case manager relationship. However, as user satisfaction with services should be understood as a process in which the working alliance, including goal agreements, actions taken, and relational bonds, are likely to change over time, user satisfaction needs to be measured at several time points to understand more fully how user satisfaction is associated with VR services.

The main strengths of the study are the horizontal perspective on user satisfaction across different VR interventions, as few rehabilitation programs include service users’ perspectives on satisfaction (47), and the rather large sample of participants with disabilities. Survey studies often poorly represent this target group, as recruitment often fails to include people with disabilities due to accessibility barriers (48). When completing the questionnaire, the participants could choose to get help with filling out the questionnaire by letting someone (their support person in the interventions or another person of their choice) read the questions and answer aloud from the other side of the table; hence, the participants were hiding their answers from the reader. As shown in Table 4, approximately 30 percent of the participants chose to be helped, which may have impacted participation but also the way the participants answered the questions, whether social influence and compliance came into play. However, as Table 4 shows, there were no significant differences in the use of help with completing the questionnaire.

Another methodological issue is that there were significantly more participants in the RCT with CM as an intervention who responded to the questionnaire. This finding might imply that persons in the two other interventions, RVR and SE, were less satisfied or indifferent to the support and intervention. If this is the case, the results from this study might be skewed.

Apart from the external missing data, there were considerable amounts of internal missing data for the variables used in the full ordinal logistic regression model. In particular, the variables “The activities help with getting a job” and Person-centeredness, which had approximately 20% internal missing data each, are worth noting. The missing data points in turn led to approximately 30% of the possible data points in the full ordinal logistic regression model being missing, which might have skewed the data. An explanation for why there were more missing data points for these two variables may be the difference in the response alternatives for the variables. The variables “The activities help with getting a job” and Person-centeredness were measured on a 4-point ordinal Likert scale; for example, the variables Satisfaction and Trust were measured on a 3-point ordinal Likert scale with more articulated response alternatives, which might have been easier to understand for persons with cognitive disabilities who were part of the sample (47).

Another methodological issue is the instability of the independent variable Trust. According to the full ordinal logistic regression model, which included the variable Trust, there were no problems violating the assumption of proportional odds; however, when Trust was tested independently, the variable violated the assumption of proportional odds to some extent. This means that the trustworthiness of the results in regard to the variable Trust might be questionable. On the other hand, on a conceptual level, one could argue that trust is indeed important for satisfaction and success with VR services (49); therefore, the results might still be valid.

## 5 Conclusions and directions for future research

The experiences of a high level of person-centeredness and trust in the support person in the VR process and the experiences of that the activities help with getting a job are more important for satisfaction among service users than the actual model of interventions in VR services. The person-centered indicators of importance to satisfaction were that the support worker listens, provides information and timely and accessible support, and plans the process together with the service users and agrees on their goals. Given that user participation is a central prerequisite in VR many services, the indicators for a person-centered approach used in this study may provide guidance on how to design services that meet individual needs and preferences for service delivery, as well as for influence over and involvement in one's own rehabilitation process. This is important for the efficiency and quality of VR services and for improving the labor market inclusion of people with disabilities.

Future research is needed to validate these findings and to study how these important factors might be implemented to an even greater extent.

## Data Availability

The datasets presented in this article are not readily available for the protection of study participant privacy. The datasets analysed during the current study are available from the corresponding author on reasonable request.
