# Supplementary material for: Analysis of factors influencing satisfaction with vocational rehabilitation services for young persons with disabilities in Sweden
Source: Front Rehabil Sci. 2025 May 30;6:1573753. doi: 10.3389/fresc.2025.1573753 (PMC12162581; doi:10.3389/fresc.2025.1573753)
Supplement: Supplementary file 1 [file Datasheet1.pdf]

Supplementary Material 1

Significance test of satisfaction with support between the three interventions.

Kruskal–Wallis Test

	Intervention	N	Mean Rank
Satisfaction with support	RVR	148	257,88
	CM	283	331,10
	SE	176	299,22
	Total	607	

	Satisfaction with support
Kruskal–Wallis H	27,319
df	2
Asymp. Sig.	<,001

a. Kruskal–Wallis Test
b. Grouping Variable: Intervention

Post hoc tests for exploring the significance of differences were performed with the Kruskal–Wallis test.

Mann–Whitney Test of RVR compared to CM

	Intervention	N	Mean Rank	Sum of Ranks
Satisfaction with support	RVR	148	182,19	26964,00
	CM	283	233,68	66132,00
	Total	431		

	Satisfaction with support
Mann–Whitney U	15938,000
Wilcoxon W	26964,000
Z	-5,194
Asymp. Sig. (2-tailed)	<,001

Mann–Whitney test of the RVR compared to the SE

	Intervention	N	Mean Rank	Sum of Ranks
Satisfaction with support	RVR	148	150,19	22227,50
	SE	176	172,86	30422,50
	Total	324		

	Satisfaction with support

Mann–Whitney U	11201,500
Wilcoxon W	22227,500
Z	-2,564
Asymp. Sig. (2-tailed)	,010

Mann–Whitney test of CM compared to SE

	Intervention	N	Mean Rank	Sum of Ranks
Satisfaction with support	CM	283	239,41	67754,00
	SE	176	214,86	37816,00
	Total	459		

	Satisfaction with support
Mann–Whitney U	22240,000
Wilcoxon W	37816,000
Z	-2,588
Asymp. Sig. (2-tailed)	,010

Supplementary Material 2

Cell information in the independent ordinal logistic regression for the independent variable “Trust in support persons”

Trust in support persons		Satisfaction with support: Bad	Quite alright	Good
No trust	Observed	1	4	2
	Expected	1,150	3,725	2,125
	Pearson Residual	-,153	,208	-,103
Partly trust	Observed	19	41	41
	Expected	13,167	51,101	36,732
	Pearson Residual	1,724	-2,010	,883
Trust	Observed	5	103	386
	Expected	11,377	95,214	387,409
	Pearson Residual	-1,913	,888	-,154

Link function: Logit.

Supplementary Material 3

Multicollinearity of the independent variables tested with the Variance Inflation Factor (VIF).

Model		Collinearity Statistics	
		Tolerance	VIF
1	(Constant)		
	Intervention	,955	1,047
	Age	,983	1,018
	Gender	,994	1,006
	Activities lead to job	,736	1,358
	Trust	,826	1,210
	Employment/Internship/Studies during intervention	,876	1,141
	Participation	,670	1,491
	Help with filling in the questionnaire	,981	1,020
